# Reaching out: junctions between cardiac telocytes and cardiac stem cells in culture

**DOI:** 10.1111/jcmm.12719

**Published:** 2015-11-05

**Authors:** Laurențiu M. Popescu, Emanuel T. Fertig, Mihaela Gherghiceanu

**Affiliations:** ^1^ Department of Advanced Studies‘Victor Babeş’ National Institute of PathologyBucharestRomania; ^2^ Department of Cellular and Molecular Medicine‘Carol Davila’ University of Medicine and PharmacyBucharestRomania; ^3^ Electron Microscopy Laboratory‘Victor Babeş’ National Institute of PathologyBucharestRomania

**Keywords:** cardiac telocytes, telopodes, cardiac stem cells, gap junctions, adherens junctions, stromal synapse

## Abstract

Telocytes (TCs) were previously shown by our group to form a tandem with stem/progenitor cells in cardiac stem cell (CSC) niches, fulfilling various roles in cardiac renewal. Among these, the ability to ‘nurse’ CSCs *in situ*, both through direct physical contact (junctions) as well as at a distance, by paracrine signalling or through extracellular vesicles containing mRNA. We employed electron microscopy to identify junctions (such as gap or adherens junctions) in a co‐culture of cardiac TCs and CSCs. *Gap junctions* were observed between TCs, which formed networks, however, not between TCs and CSCs. Instead, we show that TCs and CSCs interact in culture forming heterocellular *adherens junctions*, as well as non‐classical junctions such as *puncta adherentia* and *stromal synapses*. The stromal synapse formed between TCs and CSCs (both stromal cells) was frequently associated with the presence of electron‐dense nanostructures (on average about 15 nm in length) connecting the two opposing membranes. The average width of the synaptic cleft was 30 nm, whereas the average length of the intercellular contact was 5 μm. Recent studies have shown that stem cells fail to adequately engraft and survive in the hostile environment of the injured myocardium, possibly as a result of the absence of the pro‐regenerative components of the secretome (paracrine factors) and/or of neighbouring support cells. Herein, we emphasize the similarities between the junctions described in *co‐culture* and the junctions identified between TCs and CSCs *in situ*. Reproducing a CSC niche in culture may represent a viable alternative to mono‐cellular therapies.

## Introduction

Regenerative medicine and specifically stem cell therapies show the greatest promise in correcting the extensive ventricular scarring and massive cell loss following ischaemic events in coronary heart disease. Different cell types have been investigated and even considered for regeneration of the injured heart in animal models and humans, of which notably: embryonic stem cells(ESCs), adipose‐derived stem cells, mesenchymal stem cells, bone marrow‐derived cells and CSCs 1, 2, 3, 4, 5, 6, 7, 8, 9. Unfortunately, the results have been unconvincing and sometimes contradictory, due mainly to less than ideal cell delivery methods and poor cell engraftment and survival 10, 11, 12, 13, 14, 15, 16, 17, 18. The very low survival rate is suggested to be caused by the notoriously ‘hostile environment’ of the infarcted region, characterized by ischaemia, a heightened inflammatory response, lingering pro‐apoptotic signals and by the absence of an extracellular matrix 19, 20. In addition, successful stem cell engraftment appears to be dependent on the presence of various paracrine factors (*e.g*. growth factors, cytokines, chemokines) and extracellular vesicles, part of the so called ‘secretome’ released by stem cells and/or other neighbouring cells within CSC niches 14, 21, 22, 23, 24. It, therefore, becomes obvious that understanding the signalling mechanisms between support and stem cells, or even replicating the architecture of CSC niches *in vitro* prior to transplantation, is key for improving current therapies 25.

Among the cell types recently suggested to be involved in cardiac homoeostasis and regeneration are TCs 26, 27, 28, 29. Telocytes are distinct interstitial cells found in most organs, characterized by the presence of lengthy extensions – telopodes (Tp) 30, 31, 32, 33, 34. Indeed, our group has shown that TCs form a complex network within the myocardium, communicating with adjacent cells both through direct physical contact as well as by means of paracrine signalling 35, 36. Cardiac TCs shed at least three different types of extracellular vesicles *in situ* 36, 37 and *in culture* 38. In an experiment involving Cy5‐labelled oligoRNAs, these vesicles were successfully transferred to stem cells in culture, indicating a potential similar mechanism for the transfer of regenerative factors 39. It is plausible that stem/progenitors cells are ‘nursed’ by TCs in CSC niches, thereby sustaining a continuous cardiac renewal process in the adult mammalian heart 35, 36, 40, 41.

In this work, we demonstrate that TCs form ‘atypical’ junctions with stem cells not only in tissue but also *in culture*.

## Materials and methods

### Cell culture

Cardiac TCs were isolated from the hearts of 3‐month‐old Wistar rats and cultured as described previously 34, 39. Laboratory animals were handled in accordance with the ‘Victor Babeș’ Institute Ethics Board guidelines. Rat CSCs were a kind gift from Prof. Piero Anversa (Brigham and Women Hospital, Boston, MA, USA). This cell line is described elsewhere 42, 43.

### Transmission electron microscopy

Cells cultured for 24 and 48 hrs, were fixed and embedded in epoxy resin (Agar 100) as described previously 38. Ultra‐thin sections (~60 nm) were then obtained using a diamond knife, double stained with 1% uranyl acetate and Reynolds lead citrate and finally visualized using a Morgagni 268 TEM (FEI Company, Eindhoven, The Netherlands) at 80 kV. Digital electron micrographs were recorded using a MegaView III CCD. Image processing was done using iTEM‐SIS software (Olympus, Munster, Germany). Samples originating from tissue 41 were used for comparison.

## Results

**Figure 1:**
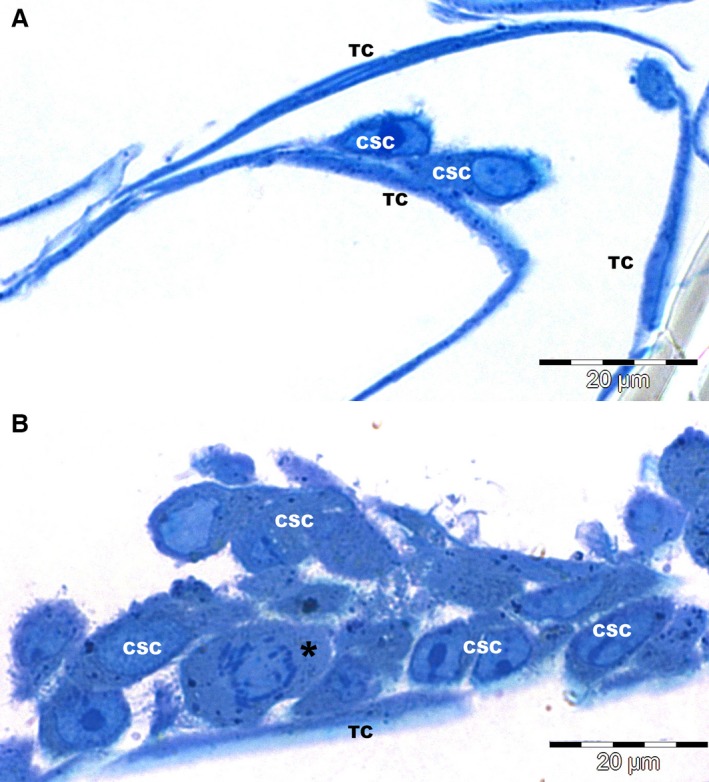
Light microscopy (1 μm semithin sections of epoxy resin embedded cells, stained with toluidine blue), reveals close contacts between cardiac stem cells (CSC) and cardiac telocytes (TC) after 24 hrs (**A**) and 48 hrs (**B**) of culture. Notably, CSCs markedly increased in number after 48 hrs of culture (one imaged cell is undergoing mitosis – *).

Telocytes were identified in culture (Fig. 1) based on the presence of very long extensions (Tp), with lengths varying from 100 to 200 μm 38. Telopodes were frequently observed in apparent contact with either CSCs or other TCs (Fig. 1). Heterocellular contacts markedly increased in number after 48 hrs of culture and this corresponded to an increase in the number of CSCs (Fig. 1B) in the presence of TCs.

**Figure 2:**
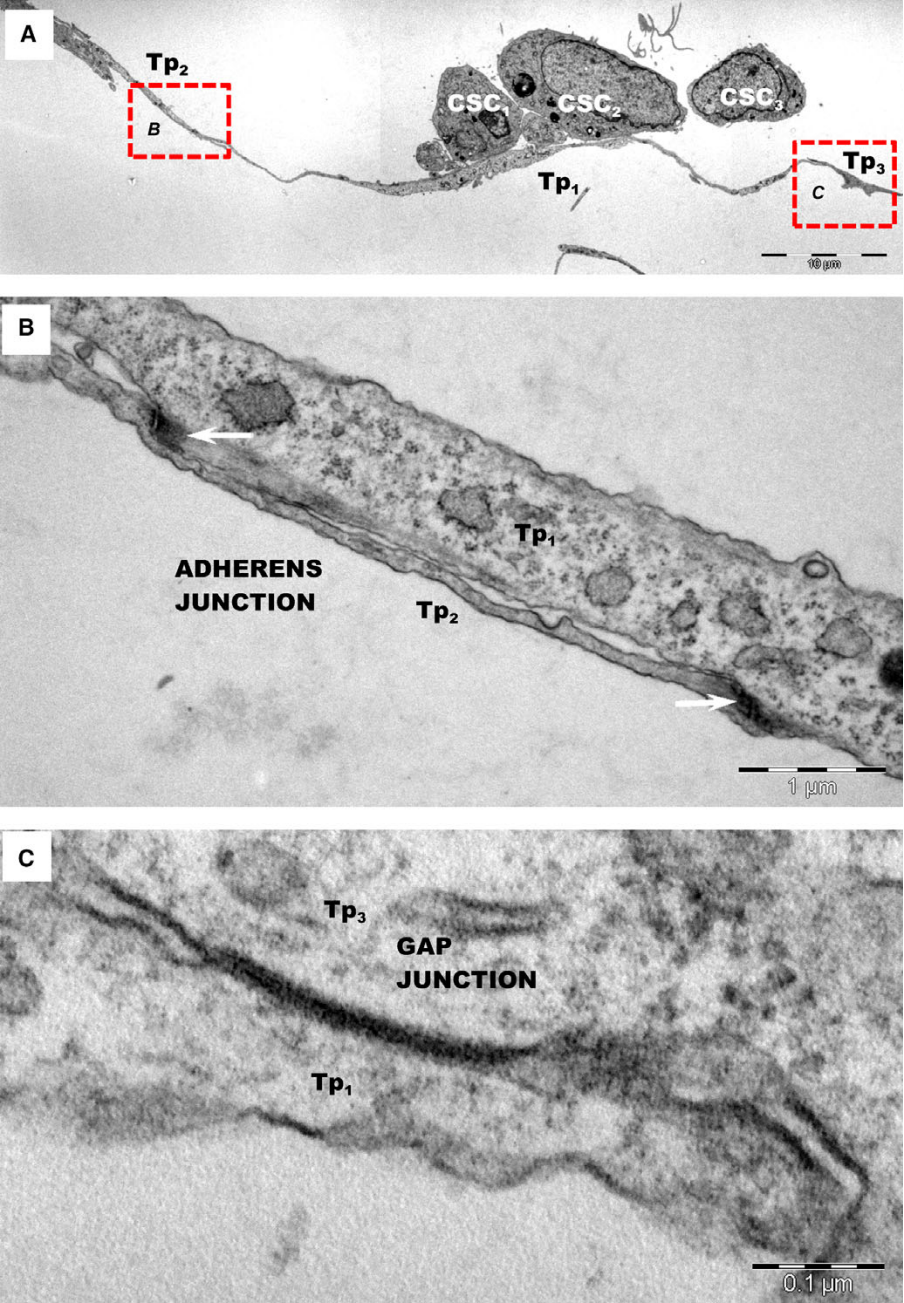
(**A**) Low magnification transmission electron microscopy images of TC–CSC after 24 hrs of culture show adherens and gap junctions in a network of telopodes (Tp1‐Tp3). (**B**) Higher magnification of AJs (white arrows) between telopodes Tp1 and Tp2 shown in image A (rectangular mark B). (**C**) Higher magnification of gap junction between Tp1 and Tp3 shown in image A (rectangular mark C), highlighting that telopodes connect through different types of junctions.

Telopodes originating from different TCs frequently formed homocellular networks, establishing both adherens (AJ) and gap junctions (GJ) at the site of contact (Fig. 2) and this was consistent with previous observations of TCs in tissue 36. Gap junctions were easily observed connecting cultured TCs to other TCs (Fig. 2C), however, not TCs to CSC. Interestingly, no GJs could be identified between TCs in the cardiac tissue.

**Figure 3:**
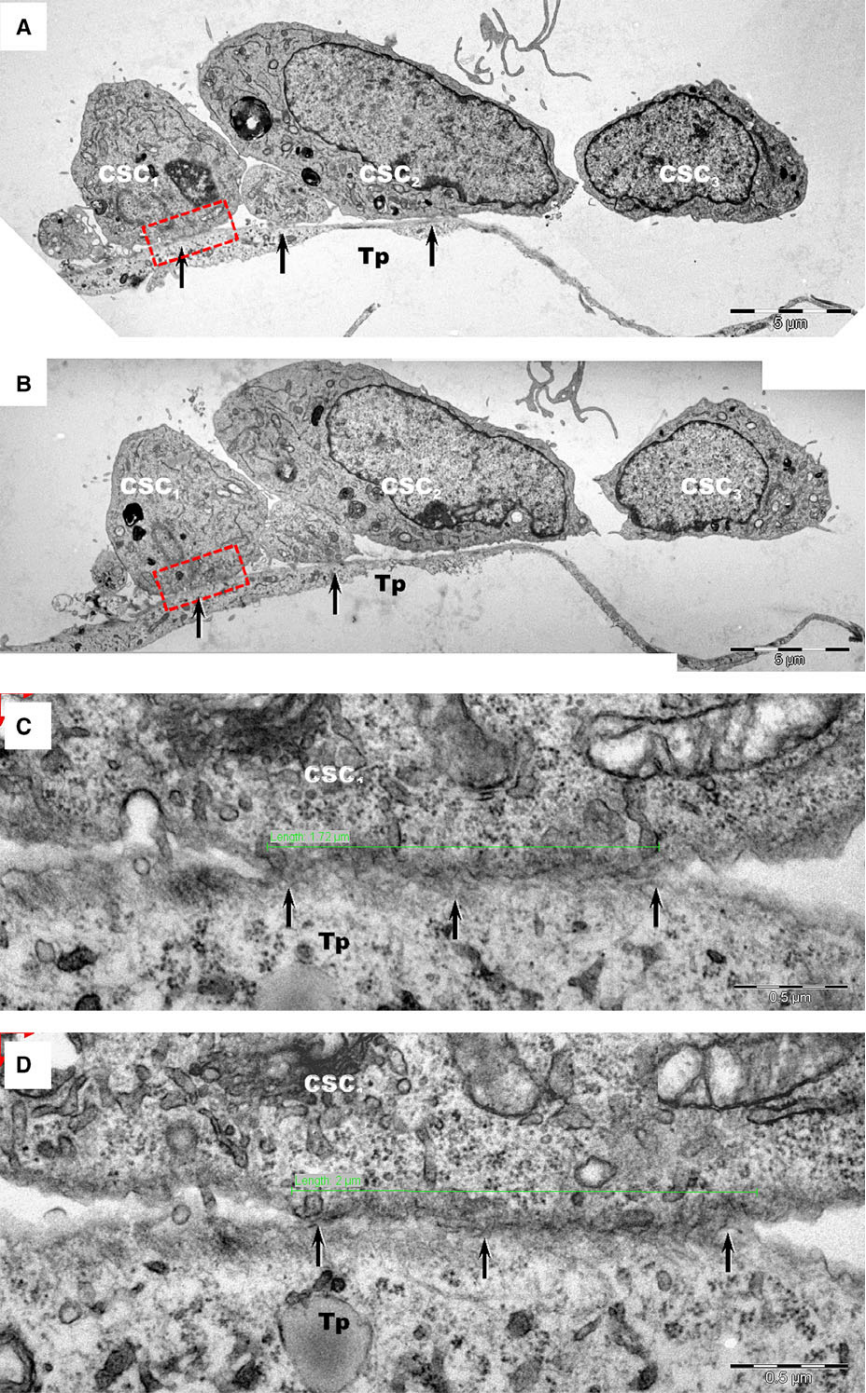
Transmission electron microscopy images of TC–CSC culture after 24 hrs. (A, B) Serial sections show close contacts (black arrows) between cardiac stem cells (CSC_1_‐CSC_3_) and telopodes (Tp) of telocytes. (**C**,** D**) Higher magnification of rectangular marked areas in images **A** and **B** highlight the interface between cardiac stem cell CSC_1_ and a telopode (Tp). An oblique sectioned *stromal synapse* (arrows) is visible between Tp and CSC_1_. The length of the stromal synapse is about 2 μm (green line). Various types of vesicles may be seen at the interface between cells.

**Figure 4:**
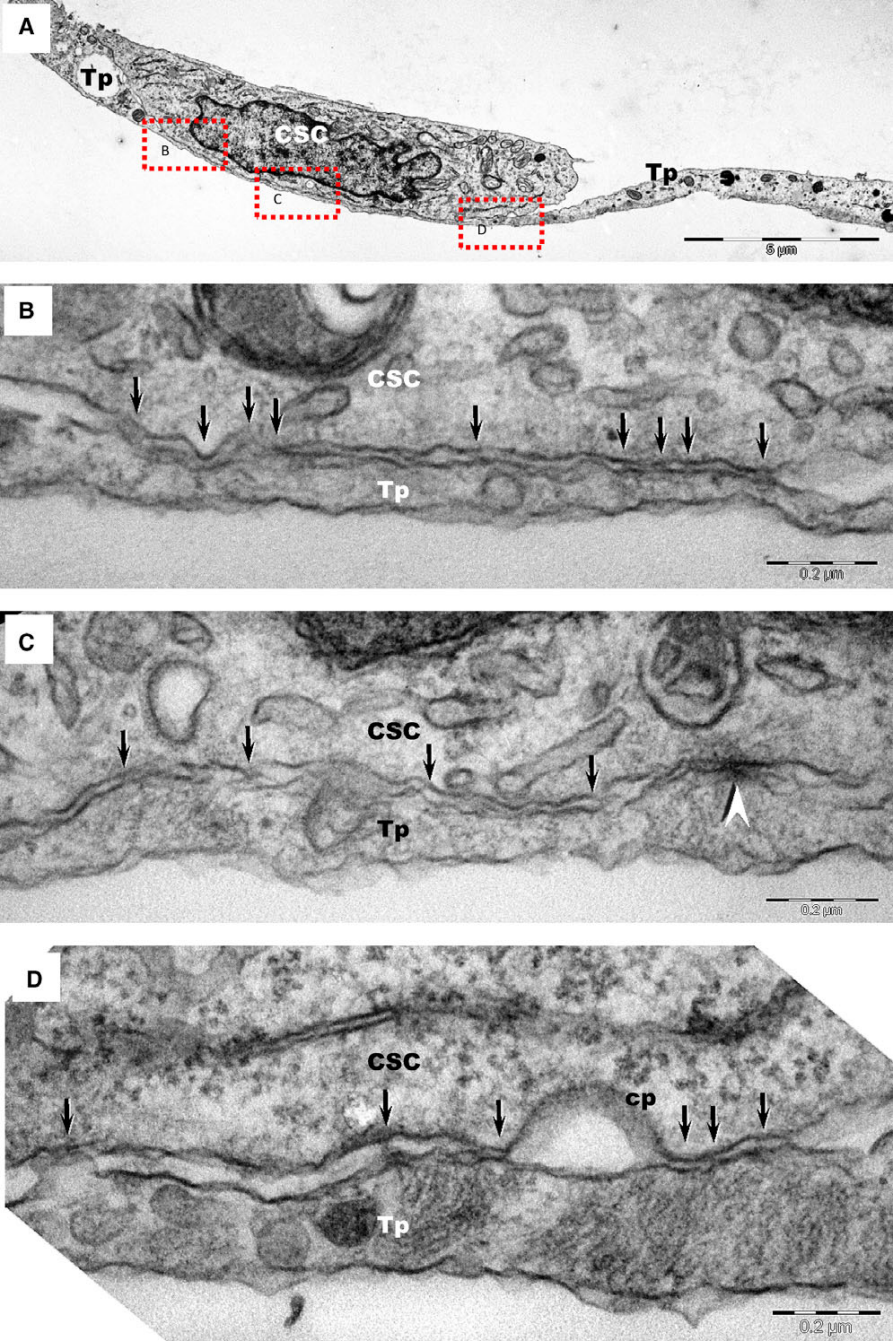
(**A**) Transmission electron microscopy images of TC–CSC culture after 48 hrs shows a telopode (Tp) in close contact with a cardiac stem cell (CSC). **(B**,** C**,** D**) Marked areas from image A are shown at higher magnification in the corresponding panels. A planar contact (*stromal synapse*) between TC and CSC can be seen associated with a number of electron‐dense structures (arrows). A *puncta adherentia* junction (arrowhead) is visible between TC and CSC in image C. Cp – coated pit.

**Figure 5:**
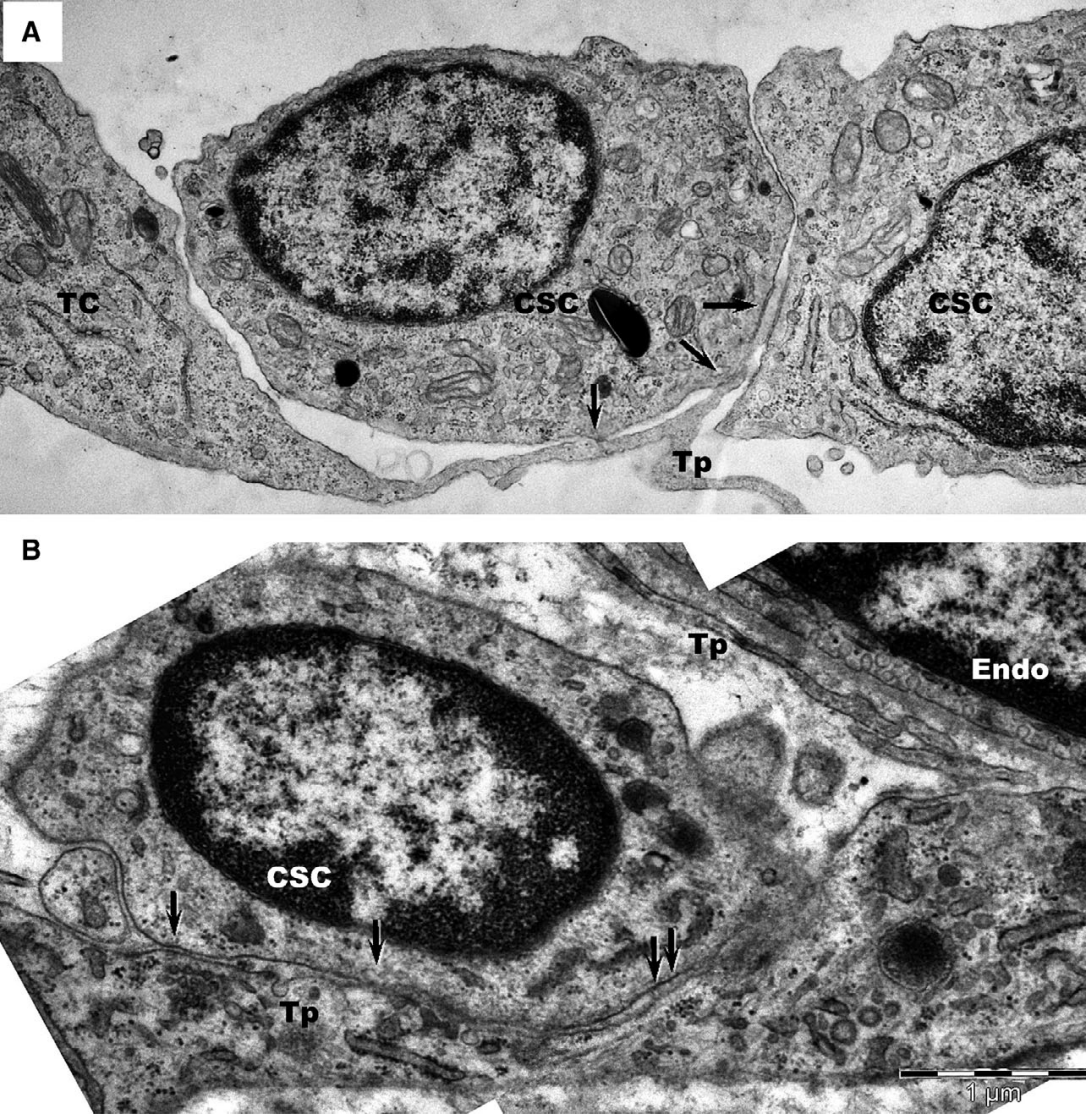
Transmission electron microscopy shows similar intercellular connections (*plain stromal synapses*) between telocytes (TC) and cardiac stem cells (CSC) *in culture* (**A**) and *in tissue* (**B**). Telopodes (Tp) connect with a cardiac stem cell (CSC) through small electron‐dense structures (arrows). Endo: endothelial cell; P: pericyte; N: nerve ending; CM: cardiomyocyte.

Telocytes formed junctions with adjacent CSCs (Figs 3, 4, 5A), identified as *stromal synapses* (Figs 3 and 4). The length of TC–CSC stromal synapse ranged from 1.8 to 12.9 μm, with a global average of 5.5 ± 5.1 μm at 24 hrs of culture (mean ± S.D.). The intercellular distance between TC–CSC at the interface (synaptic cleft) varied between 9.9 and 58.8 nm, with an average of 30.6 ± 12.8 nm (mean ± S.D.). Numerous electron‐dense nanostructures could be observed in the synaptic cleft between TCs and CSCs (Figs 4B–D and 5A). These structures had a minimum length of 7.9 nm and a maximum length of 24 nm, with an average of 14.8 ± 4.5 nm (mean ± S.D.).

The *Puncta adhaerentia* type junction was infrequently found connecting TCs and CSCs (Fig. 4C). Telocytes and CSCs also maintained contact by means of paracrine signalling, *via* different types of extracellular vesicles (Fig. 5A), however, these are described elsewhere 38.

**Figure 6:**
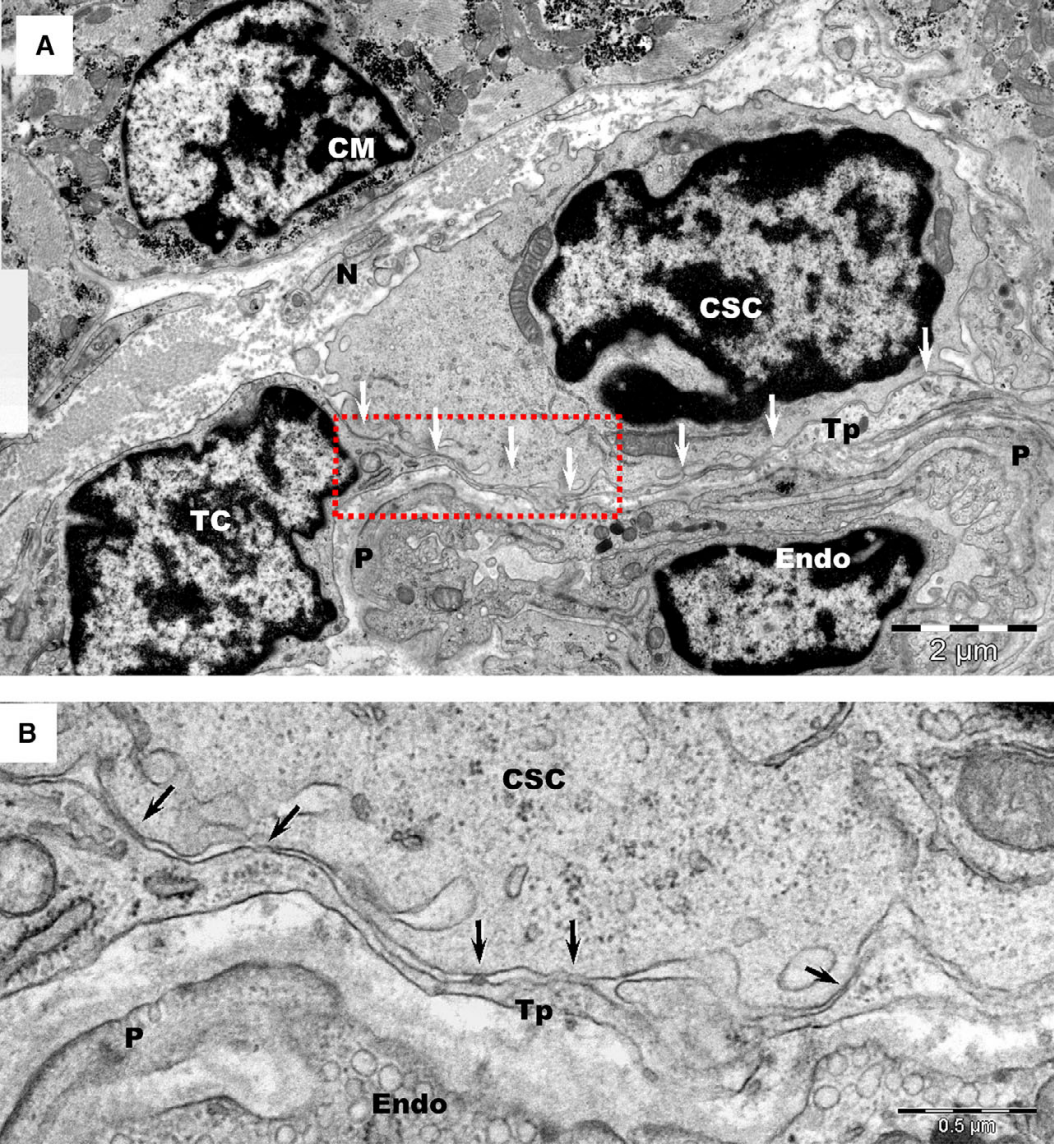
(**A**) Transmission electron microscopy of human atrial tissue represents a glimpse into the complex environment of the cardiac stem cell niche, which comprises: telocytes (TC), cardiac stem cells (CSC), capillaries (Endo: endothelial cell; P: pericytes) and nerve endings (N). CM – adult cardiomyocyte. Arrows indicate the close contacts between a telopode (Tp) and a CSC. (**B**) Higher magnification of the rectangular marked area in image **A**.

**Figure 7:**
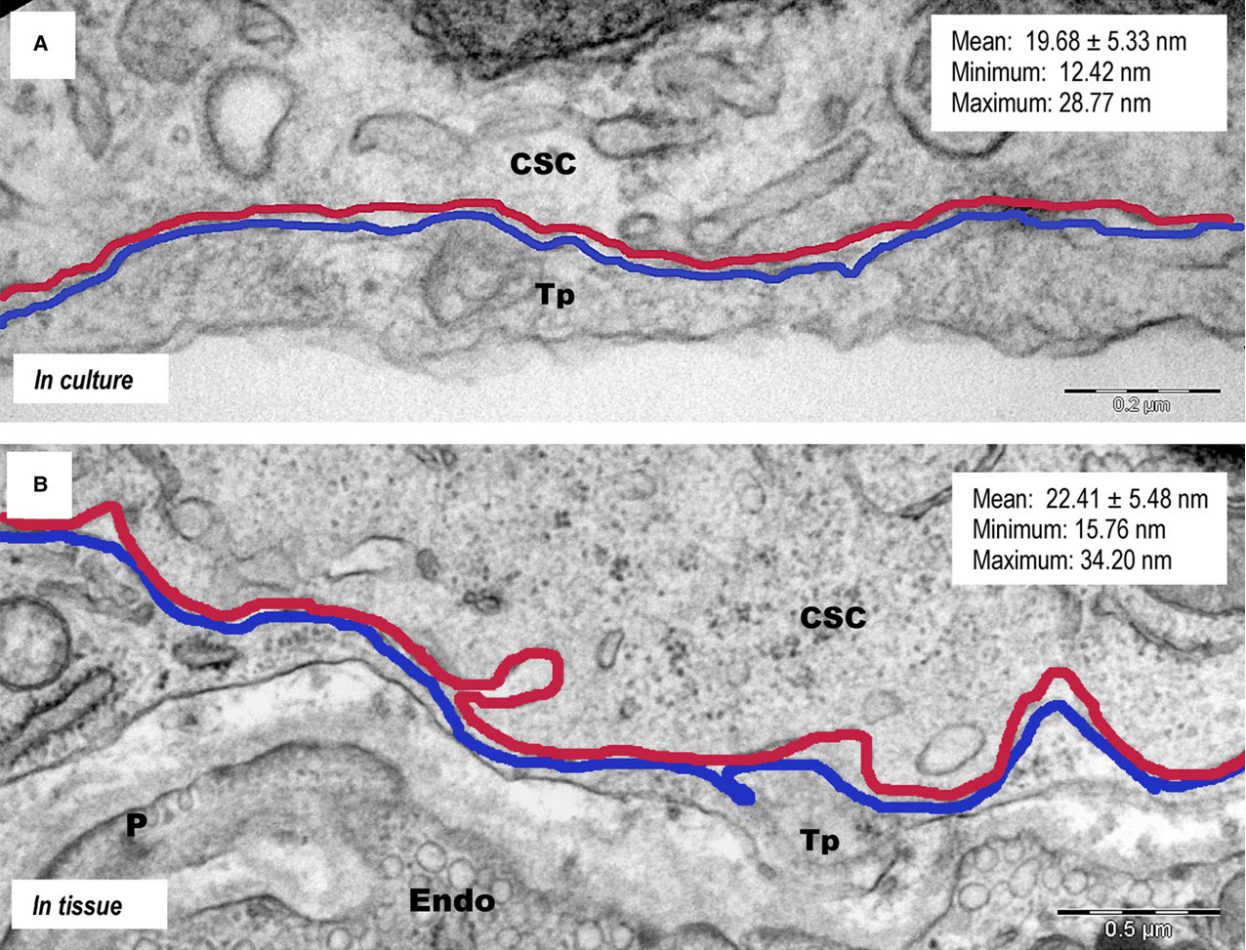
Schematic representation of *plain stromal synapses* between the cell membranes of telopodes (Tp) and those of cardiac stem cells (CSC) *in culture* (**A**) and *in tissue* (**B**), the former spanning a distance of approximately 20 nm.

Intercellular contacts between TCs and CSCs were also analysed in tissue samples for comparison. The stromal synapse associated with electron‐dense nanostructures (Figs 5B and 6) was the most common type of junction found in tissue. The intercellular distances in tissue were between 20 and 30 nm and the minimum distance between the cellular membranes was approximately 15 nm. Contours drawn over the opposing cellular membranes emphasize the similarities of heterocellular junctions between the TCs and CSCs in culture and in tissue (Fig. 7).

## Discussion

Here, we confirm that TCs display a similar morphology *in culture* as they do *in tissue* and form the same types of cell‐to‐cell junctions between themselves, as well as with CSCs, supporting the idea of an active involvement in the homoeostasis of cardiac tissue. This study suggests that replicating the architecture of CSC niches *in vitro* may be possible.

**Figure 8:**
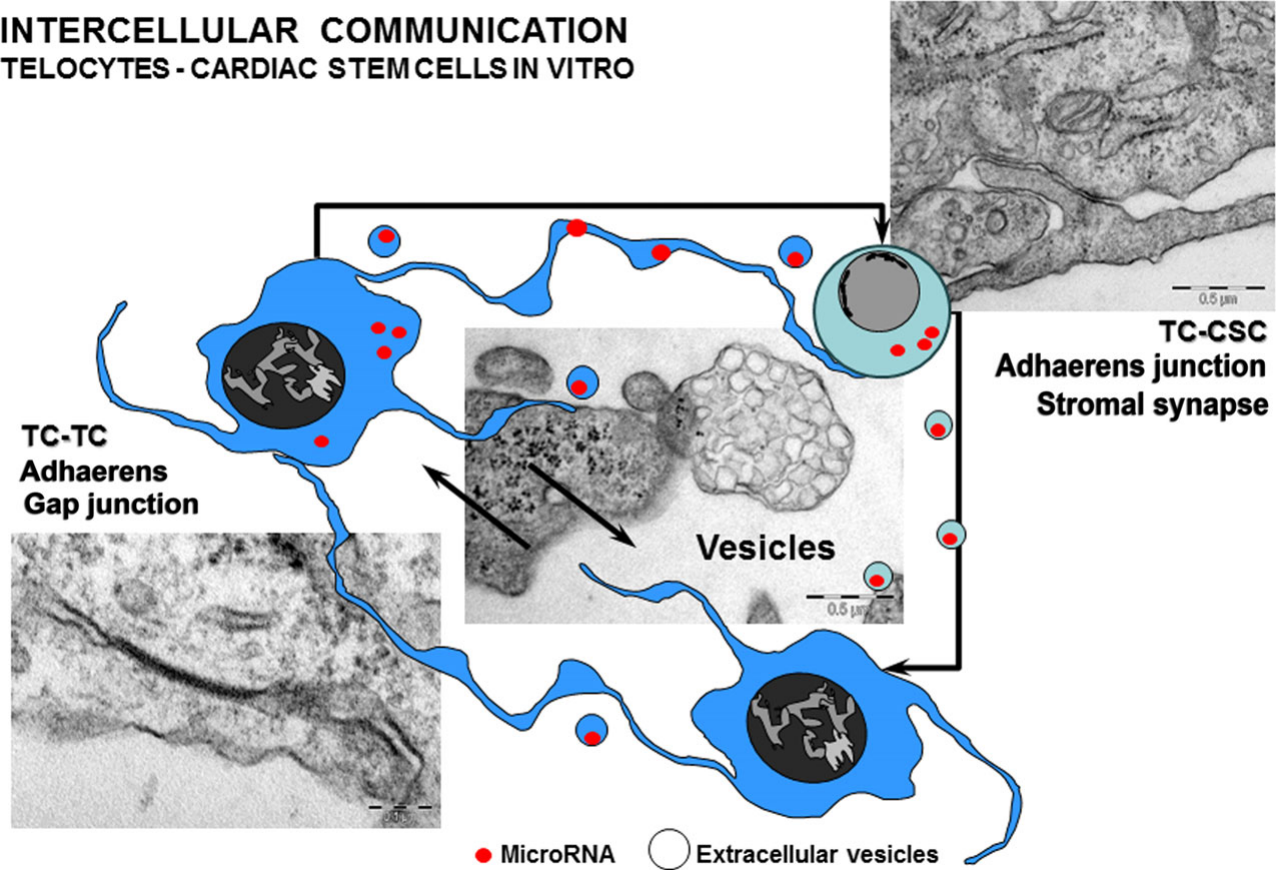
Schematic representation highlighting the different types of intercellular communication in TC‐CSC cultures. (modified with permission from 39).

In tissue, TCs may be identified as a result of their peculiar morphology, with Tp forming vast cellular networks, and these characteristics were exhaustively discussed in a previous review from our group 31. In cardiac tissue especially, TCs were suggested to function as ‘nurse‐cells’ facilitating the maturation of cardiac stem/progenitor cells 35, 36, 40. However, although recent cell‐culture data indicated that TCs communicate over long distances with stem cells through extracellular vesicles 39, the direct contacts between TCs and other cell types in culture remained largely unexplored. Understanding cardiac heterocellular communication (Fig. 8), both direct 36 and through paracrine signalling 38, 39 may prove essential for successful cell‐based therapies.

Telocytes formed homocellular networks by means of GJs, however, no such junctions could be seen between TCs and CSCs *in culture*. This may have been the result of the relatively short culture times, but more likely represents a characteristic of the partnership between the two types of cells. Similarly, embryonic fibroblast feeder cells were not found to form GJs with human ESCs and mouse ESCs 44. In fact, heterocellular GJs are not normally seen between different cell types. Contrary to a previous report indicating that fibroblasts establish GJs with cardiomyocytes in tissue and in culture 45, our group has not been able to visualize such junctions between TCs and cardiomyocytes in tissue 36, reinforcing the idea that GJs are restricted to homocellular communication.

Telocytes formed both homocellular, as well as heterocellular AJs with CSCs. Classic AJs formed between stem and support cells in epithelial and stromal niches were previously shown to control the geometry of division, by facilitating the proper positioning of centrosomes 46. As we have seen an increase in the number of CSCs in the presence (but not the absence) of cardiac TCs, it could be speculated that similar mechanisms take place in CSC‐TC co‐cultures, however, this hypothesis warrants further study. *Puncta adhaerentia*, consisting of cadherin–catenin clusters, are found at cell–cell contacts often during early stages of AJ assembly 47 and such junctions were also seen in the case of TC–CSC cultures.

Additionally, previous studies from our group 36, 48 showed that heterocellular contacts in tissue can occur by means of stromal synapses and these were also observed in TC–CSC co‐cultures. Stromal synaptic regions, akin to immune synapses 49, 50, are defined by intercellular distances within the molecular interaction range (15–100 nm), allowing the presence of a gap (synaptic cleft) in which receptor–ligand interactions occur. Consistent with the observations of TCs and CSCs or cardiomyocytes in tissue 36, 48, 51, electron‐dense nanostructures connecting the two apposing membranes over distances shorter than 25 nm were frequently associated with these types of junctions. The molecular composition of these nanostructures is unknown.

The exact role of TCs in cardiac tissue regeneration remains an elusive, but desirable target, with potential applications in stem cell therapy. Future in‐depth studies of the molecular components of stromal synapse and the dynamics of these components in normal and diseased tissues will shed a new light on the signalling mechanisms between stem and support cells (Fig. 8). This, in turn, may help overcome the current limitations of stem cell engraftment in myocardial tissue following ischaemic events.

## Conflicts of interest

The authors confirm that there are no conflicts of interest.
